# Along for the Ride: Intrahepatic Cholangiocarcinoma with Concomitant LECT2 Amyloidosis

**DOI:** 10.1155/2020/8830763

**Published:** 2020-07-16

**Authors:** Phoenix D. Bell, Aaron R. Huber, Tom C. DeRoche

**Affiliations:** ^1^University of Rochester Medical Center, Rochester, NY, USA; ^2^Kaiser Permanente Northwest, Portland, OR, USA

## Abstract

We present a case of a 69-year-old Hispanic male with a past medical history of type II diabetes mellitus who presented with a two-month history of abdominal pain. A CT scan was performed which identified a liver mass. Biopsy of the liver mass revealed infiltration of normal liver parenchyma by atypical glands surrounded by pale eosinophilic material. The atypical glands were positive for CK7, while negative for CK20, CDX-2, and TTF-1, consistent with intrahepatic cholangiocarcinoma. A Congo red stain was performed, which highlighted salmon-orange areas, some with a globular appearance, around the glands and within the sinusoids and vasculature. Under polarized light, these areas displayed apple-green birefringence. These findings were consistent with amyloidosis, which was further supported by identification of ALECT2- (leukocyte chemotactic factor-2-) type amyloid on mass spectrometry. To our knowledge, this is the first documented case of intrahepatic cholangiocarcinoma arising in association with LECT2 amyloidosis.

## 1. Introduction

Systemic amyloidosis is a disease caused by the extracellular deposition of abnormal amyloid protein fibrils in tissues, frequently resulting in organ dysfunction or failure. Over 30 types of amyloid are recognized, with the most common types being immunoglobulin light chain (AL) and serum amyloid A (SAA) amyloidosis [1]. In 2008, Benson et al. [2] discovered leukocyte chemotactic factor 2 (LECT2) amyloid, which is now recognized as the third most common cause of systemic amyloidosis [3, 4]. LECT2 amyloidosis is most commonly seen in Hispanic patients with progressive renal failure; however, the etiology is unknown [5, 6]. LECT2 amyloid most commonly deposits in the kidneys and the liver [7]. Histologically, AL and SAA hepatic amyloidosis cannot be distinguished based upon their deposition patterns [8]; however, it has been suggested that LECT2 amyloidosis can be identified by its globular appearance [9]. Herein, we report a case of intrahepatic cholangiocarcinoma associated with LECT2 amyloidosis. We aim to further characterize the histologic findings in LECT2 hepatic amyloidosis and we emphasize the importance of correctly subtyping LECT2 amyloid to avoid exposing patients to unnecessary therapy.

## 2. Case Presentation

**Figure 1:**
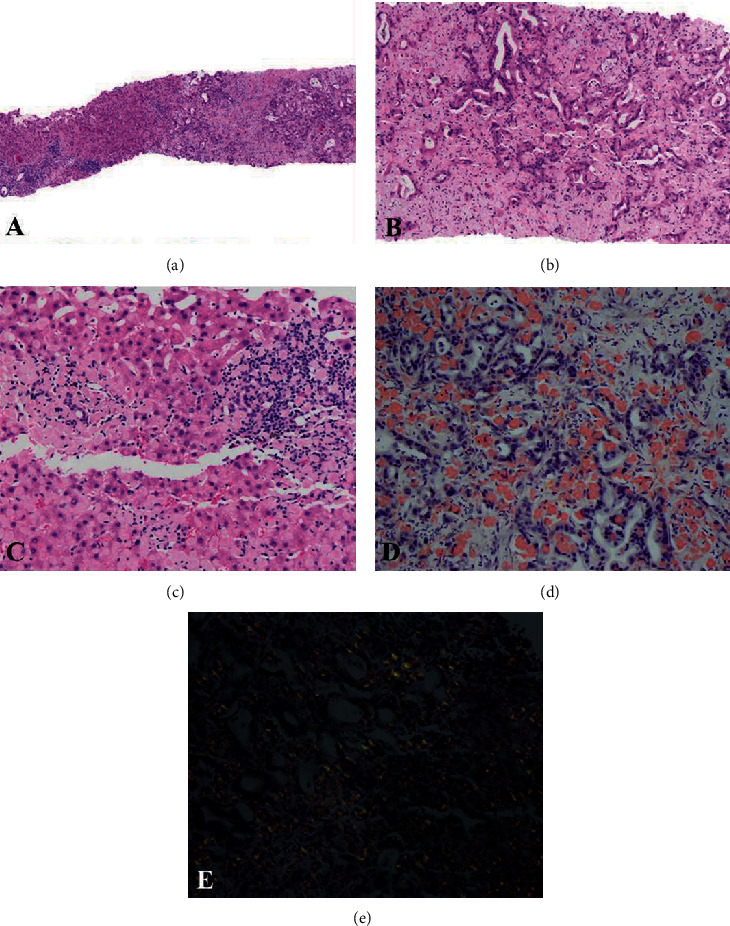
Liver biopsy (a, b) infiltration of normal hepatic parenchyma by irregular glands with surrounding eosinophilic material (H&E; (a) 40x, (b) 100x); (c) eosinophilic material with a globular morphology (H&E, 200x); (d) eosinophilic material appearing salmon-orange on Congo red stain (Congo red, 100x); (e) eosinophilic material with apple-green birefringence under polarized light (Congo red, polarized, 100x).

A 69-year-old Hispanic male with a history of type II diabetes mellitus presented to the emergency department with a two-month history of worsening abdominal pain. A computed tomography (CT) scan was ordered, which revealed a 4.3 cm ill-defined, low-density mass in segment 5 of the liver with nonspecific soft tissue encasing the superior mesenteric artery. As per the radiologist's impression, the mass was adjacent to, but did not originate from, the pancreas. No additional sites of disease were seen on a subsequent positron emission tomography (PET) scan. The patient was referred for a liver mass biopsy. At low magnification, the biopsy revealed almost complete destruction of the hepatic parenchyma by irregular glands surrounded by fibrous tissue (Figure 1(a)). Medium power showed dense pale eosinophilic material separating angulated glands lined by cells with a moderate amount of deeply eosinophilic cytoplasm with round to ovoid hyperchromatic nuclei (Figure 1(b)). The pale eosinophilic material was also present within the sinusoids of uninvolved hepatocytes (Figure 1(c)). A Congo red stain was performed, which showed salmon-orange areas around the glands and within the sinusoids and vasculature, some of which showed a globular appearance (Figure 1(d)). These areas displayed apple-green birefringence under polarized light and surrounded the glands and involved vessel walls (Figure 1(e)). These findings were consistent with amyloidosis, and the biopsy was sent for mass spectrometry analysis, which revealed a peptide profile consistent with ALECT2- (leukocyte chemotactic factor-2-) type amyloid. The glands were positive for CK7, while negative for CK20, CDX-2, and TTF-1. Given the tumor morphology and CK7 positivity, hepatocellular carcinoma was excluded and no mucin stain was performed. The patient was diagnosed with intrahepatic cholangiocarcinoma (ICC) with hepatic LECT2 amyloidosis.

One month after diagnosis, the patient was started on palliative gemcitabine and cisplatin. After three months of treatment, his CA19-9 decreased from 284 to 103 and the size of the mass decreased from 4.2 cm to 3 cm; however, the degree of vascular encasement worsened. No treatment was given for the amyloidosis. Currently, the patient is alive and tolerating treatment.

## 3. Discussion

We have presented the first documented case of ICC associated with LECT2 hepatic amyloidosis. LECT2 amyloidosis is the third most common cause of systemic amyloidosis, after AL and SAA [1, 2]. LECT2 is most commonly found in Hispanic patients of Mexican descent but has also been documented in First Nations People from Canada, Native Americans, and Punjabis [4, 6, 10]. The prevalence is unknown as patients present with progressive renal failure and kidney biopsy is not part of routine management [10]. The etiology of LECT2 amyloidosis is unknown; however, in one study of 40 patients with LECT2 amyloidosis, 50% and 38% of patients had a past medical history of hypertension or diabetes mellitus, respectively [5]. LECT2 most commonly affects the kidneys followed by the liver but may also be found in the colon, spleen, or adrenal glands [2, 6].

Patients with systemic amyloidosis usually have liver involvement. In the clinical setting, patients with hepatic amyloidosis may present with hepatomegaly, ascites, cholestasis, jaundice, or portal hypertension [11–14]. Liver biopsies show sinusoidal and intravascular amyloid deposition for both AL and AA amyloidosis; thus, the deposition pattern cannot distinguish AL from AA amyloid [8]. Interestingly, Chandan et al. [9] analyzed 24 cases of LECT2 hepatic amyloidosis, and 100% of cases showed globular hepatic amyloid deposition in the hepatic sinusoids and portal tracts. Our findings are consistent with this report; therefore, the presence of a globular pattern of hepatic amyloid deposition should prompt consideration of LECT2 amyloidosis, which can be confirmed by LECT2 immunopositivity and, in more difficult cases, mass spectrometry.

Treatment for amyloidosis depends upon the subtype of amyloid that is being depositing in the tissues. In AL amyloidosis, the underlying cause of light chain deposition is vfrequently due to a plasma cell dyscrasia, thus these patients are treated with chemotherapy or autologous stem cell transplant [15, 16]. Inflammatory states contribute to the development of SAA amyloidosis; thus, these patients are treated with medications targeted towards the specific underlying cause of inflammation [17], which may range from antibiotics to biologics [3]. In more advanced cases, as is seen in patients with hereditary amyloidosis, liver transplant may be the only treatment [18]. In contrast, the etiology of LECT2 amyloidosis is unknown and there is currently no treatment. Similar to most other types of amyloidosis, patients with LECT2 amyloidosis often develop renal failure; thus, closely monitoring a patient's renal function and volume status is an important part of management. Further, once a patient develops end-stage renal disease (ESRD), kidney transplant may be considered [7]. It is important to note, that although the prognosis of LECT2 amyloidosis is uncertain, patients with LECT2 amyloidosis do have a better prognosis than those with AL amyloidosis due to lack of substantial cardiac involvement [19].

The main histologic differential diagnosis for amyloidosis is light chain deposition disease (LCDD), although this is rare in the liver. Like AL amyloidosis, LCDD is commonly seen in patients with plasma cell dyscrasias and is caused by the deposition of monoclonal immunoglobulin light chains in tissues, most commonly the kidneys [20]. It differs from amyloidosis in that the immunoglobulins are characterized by nonorganized electron-dense granular deposits, rather than organized *β*-pleated sheets [21]. Similar to amyloidosis, LCDD is histologically characterized by the presence of amorphous eosinophilic material that can distort normal hepatic architecture. A Congo red stain can distinguish these two entities: LCDD is negative, while amyloidosis is positive [20].

## 4. Conclusion

In summary, we present the first documented case of intrahepatic cholangiocarcinoma with concomitant hepatic LECT2 amyloidosis. Consistent with prior reports, our patient is Hispanic with a history of longstanding renal disease. Additionally, if our patient did not have symptoms associated with cholangiocarcinoma, the diagnosis of amyloidosis would have been delayed further. Histologically, our case demonstrates globular amyloid deposition, both within the normal liver parenchyma and associated with the carcinoma—a pattern that may be unique to LECT2. Lastly, we stress the importance of identifying patients with LECT2 amyloidosis so as to avoid unnecessary therapies, such as chemotherapy, anti-inflammatory medications, or liver transplant, which are used in AL, AA, and hereditary amyloidosis, respectively [3].

## References

[1] Sipe J. D., Benson M. D., Buxbaum J. N. (2014). Nomenclature 2014: amyloid fibril proteins and clinical classification of the amyloidosis. *Amyloid*.

[2] Benson M. D., James S., Scott K., Liepnieks J. J., Kluve-Beckerman B. (2008). Leukocyte chemotactic factor 2: a novel renal amyloid protein. *Kidney International*.

[3] Wechalekar A. D., Gillmore J. D., Hawkins P. N. (2016). Systemic amyloidosis. *Lancet*.

[4] Larsen C. P., Walker P. D., Weiss D. T., Solomon A. (2010). Prevalence and morphology of leukocyte chemotactic factor 2-associated amyloid in renal biopsies. *Kidney International*.

[5] Larsen C. P., Kossmann R. J., Beggs M. L., Solomon A., Walker P. D. (2014). Clinical, morphologic, and genetic features of renal leukocyte chemotactic factor 2 amyloidosis. *Kidney International*.

[6] Murphy C. L., Wang S., Kestler D. (2010). Leukocyte chemotactic factor 2 (LECT2)-associated renal amyloidosis: a case series. *American Journal of Kidney Diseases*.

[7] Nasr S. H., Dogan A., Larsen C. P. (2015). Leukocyte cell-derived chemotaxin 2-associated amyloidosis: a recently recognized disease with distinct clinicopathologic characteristics. *Clinical Journal of the American Society of Nephrology*.

[8] Buck F. S., Koss M. N. (1991). Hepatic amyloidosis: morphologic differences between systemic AL and AA types. *Human Pathology*.

[9] Chandan V. S., Shah S. S., Lam-Himlin D. M. (2015). Globular hepatic amyloid is highly sensitive and specific for LECT2 amyloidosis. *The American Journal of Surgical Pathology*.

[10] Larsen C. P., Ismail W., Kurtin P. J., Vrana J. A., Dasari S., Nasr S. H. (2016). Leukocyte chemotactic factor 2 amyloidosis (ALECT2) is a common form of renal amyloidosis among Egyptians. *Modern Pathology*.

[11] Mohr A., Miehlke S., Klauck S., Röcken C., Malfertheiner P. (1999). Hepatomegaly and cholestasis as primary clinical manifestations of an AL-kappa amyloidosis. *European Journal of Gastroenterology & Hepatology*.

[12] Damlaj M., Amre R., Wong P., How J. (2014). Hepatic ALECT-2 amyloidosis causing portal hypertension and recurrent variceal bleeding: a case report and review of the literature. *American Journal of Clinical Pathology*.

[13] Ford M., Disney B., Shinde V., Ishaq S. (2018). Hepatic amyloidosis: a cause of rapidly progressive jaundice. *BMJ Case Reports*.

[14] Dias T., Ferreira D., Moreira H., Nascimento T., Santos A., Carvalho A. (2019). A case of severe cholestasis due to hepatic AL amyloidosis. *GE Portuguese Journal of Gastroenterology*.

[15] Palladini G., Perfetti V., Obici L. (2004). Association of melphalan and high-dose dexamethasone is effective and well tolerated in patients with AL (primary) amyloidosis who are ineligible for stem cell transplantation. *Blood*.

[16] Cibeira M. T., Sanchorawala V., Seldin D. C. (2011). Outcome of AL amyloidosis after high-dose melphalan and autologous stem cell transplantation: long-term results in a series of 421 patients. *Blood*.

[17] Lachmann H. J., Goodman H. J. B., Gilbertson J. A. (2007). Natural history and outcome in systemic AA amyloidosis. *The New England Journal of Medicine*.

[18] Sattianayagam P., Gibbs S. D., Pinney J. H. (2011). The role of liver transplantation in hereditary non-neuropathic systemic amyloidosis. *Gastroenterology*.

[19] Dogan A. (2017). Amyloidosis: insights from proteomics. *Annual Review of Pathology: Mechanisms of Disease*.

[20] Ronco P. M., Alyanakian M. A., Mougenot B., Aucouturier P. (2001). Light chain deposition disease: a model of glomerulosclerosis defined at the molecular level. *Journal of the American Society of Nephrology*.

[21] Urowitz M. B., Bookman A. A. M., Koehler B. E., Gordon D. A., Smythe H. A., Ogryzlo M. A. (1976). The bimodal mortality pattern of systemic lupus erythematosus. *The American Journal of Medicine*.

